# Preoperative Imaging Risk Findings for Postoperative New Stroke in Patients With Acute Type A Aortic Dissection

**DOI:** 10.3389/fcvm.2020.602610

**Published:** 2020-11-30

**Authors:** Hongliang Zhao, Fan Guo, Jingji Xu, Yuanqiang Zhu, Didi Wen, Weixun Duan, Minwen Zheng

**Affiliations:** ^1^Department of Radiology, Xijing Hospital, Fourth Military Medical University, Xi'an, China; ^2^Department of Cardiovascular Surgery, Xijing Hospital, Fourth Military Medical University, Xi'an, China

**Keywords:** acute type A aortic dissection, computed tomography angiography, diffusion-weighted magnetic resonance imaging, risk factors, stroke

## Abstract

**Background:** Stroke is a common postoperative complication in patients with acute type A aortic dissection (ATAAD). We aimed to explore the preoperative imaging risk findings for postoperative new stroke in patients with ATAAD.

**Methods:** From January 2015 to December 2018, 174 patients with ATAAD who underwent preoperative aortic computed tomography angiography (CTA) and cerebral diffusion-weighted imaging (DWI) as well as postoperative brain CT were included, and divided into DWI (+) and DWI (–) groups. Pre- and intraoperative variables were collected, and logistic regression analysis was used to determine the independent risk predictors of postoperative new stroke.

**Results:** The incidence of postoperative new stroke was 18.4% (32/174) in patients with ATAAD. Postoperative stroke was detected in 13 (31.0%) patients in the DWI (+) group and in 19 (14.4%) patients in the DWI (–) group with significant difference (*P* = 0.016). In the DWI (+) group, the lesions of the cerebral infarction located in the unilateral cerebral hemisphere and distributed more than three lobes (*P* = 0.007) were an independent risk factor for postoperative new stroke. Hypotension (*P* = 0.002), retrograde ascending aorta dissection with thrombosis of the false lumen (*P* = 0.010), aortic arch entry (*P* = 0.035), and coronary artery involvement (*P* = 0.001) were independent risk factors for postoperative stroke in the DWI (–) cohort.

**Conclusions:** Patients with ATAAD with cerebral infarction are more likely to develop postoperative new stroke; thus, a preoperative DWI examination may be necessary. DWI lesions distributed more than 3 lobes in the unilateral hemisphere suggest a high possibility of postoperative stroke. For patients with ATAAD with normal brain, particular attention should be given to the CTA findings of false lumen thrombosis, aortic arch entry, and coronary artery involvement to avoid postoperative stroke.

## Introduction

Stroke, as a critical postoperative complication after emergent surgery for acute type A aortic dissection (ATAAD), is associated with higher hospital mortality, longer hospital stays, and persistent neurological impairment (1). Despite numerous innovations in contemporary perioperative anesthetic and surgical management, stroke after surgical treatment of ATAAD remains high, ranging from 2.9 to 30.4% in published series (2–8).

Previous reports described that preoperative cerebral malperfusion in patients with ATAAD is a predictor for postoperative stroke and detrimental outcome (9–11). Cerebral malperfusion was diagnosed if patients with ATAAD had neurological symptoms and signs with ultrasonography or radiographic evidence of dissection of the corresponding aortic branch vessel (12). However, a major drawback of this definition is the absence of direct intracranial imaging evidence such as magnetic resonance imaging (MRI) demonstration of diffusion-weighted imaging (DWI) abnormalities. Imaging evidence of dissection in aortic arch branch vessels does not necessarily correspond to cerebral malperfusion (13). This variation perhaps can be explained by failure to record a detailed neurological examination in critically ill patients leading to an underestimation of neurological complications, particularly for an emergency physician or cardiovascular surgeon. This may lead to inaccurate predictions of postoperative stroke in patients with ATAAD.

Aortic computed tomography angiography (CTA) is the first choice for aortic dissection, and DWI enables identification of ischemic injury even in the hyperacute stage in the absence of or before structural changes become evident on conventional brain MRI. Although numerous risk factor constellations have been evaluated (2, 14–16), the predictors were primarily concerned with preoperative status and intraoperative management information of patients with ATAAD. Imaging evidence is commonly overlooked, and the accurate correlation between preoperative imaging findings of the brain, neck, and aorta and postoperative neurological complications in patients with ATAAD is rarely discussed. Whether providing accurate imaging evidence would lead to better management strategy and improved postoperative efficiency in patients with ATAAD is also unknown. Identifying preoperative imaging findings and features related to postoperative new stroke is clinically important in patients with ATAAD. In this study, we aimed to explore preoperative aortic and carotid CTA findings as well as DWI characteristics related to the risk in predicting postoperative new stroke in patients with ATAAD.

## Materials and Methods

### Patients

This retrospective study complied with the Helsinki Declaration (2000) and was approved by the institutional review board of Xijing Hospital affiliated with the Fourth Military Medical University (20120216-4). From an internal database, we retrospectively reviewed all patients with ATAAD who underwent standard procedure surgery from January 2015 to December 2018. ATAAD diagnosis was confirmed with CTA and was defined as any non-traumatic dissection of the aorta proximal to the left subclavian artery presenting within 14 days after symptom onset. The inclusion criteria were as follows: (1) available preoperative aortic CTA data; (2) available preoperative DWI data; and (3) available postoperative brain CT data. Exclusion criteria included (1) previous aortic surgical history (*n* = 16); (2) history of old cerebral infarction or hemorrhagic stroke (*n* = 7); (3) unevaluable CTA images (*n* = 7); (4) interval from admission to surgery of >24 h (*n* = 31); (5) without preoperative DWI examination (*n* = 76); and (6) postoperative non-neurogenic death (*n* = 12). A total of 149 patients were excluded. Finally, 174 eligible patients with ATAAD were included in this study. Patient demographics, previous medical history, preoperative status, imaging data, and intraoperative and postoperative data were reviewed and recorded. Preoperative hypotension was defined as systolic blood pressure <90 mmHg or requiring catecholamines to maintain blood pressure for any reason. In view of the heterogeneity of the assessment of neurological symptoms and signs, all 174 patients were divided into DWI (+) or DWI (–) group based on preoperative DWI imaging. DWI (+) was defined as one or more non-contiguous hyperintense lesions on brain DWI images. Patients in each group were further divided into two subgroups: postoperative stroke or no stroke. Postoperative new stroke was defined as the presence of neurological symptoms (NS) and signs and new cerebral infarction on CT during hospital stay in patients with ATAAD.

### Imaging

#### Preoperative Aortic CT Scans and Postoperative Brain CT

All examinations were performed using second-generation dualsource CT scanner (Somatom Definition Flash; Siemens Healthcare, Forchheim, Germany) with a high-pitch spiral scan mode. Patients underwent combined CTA imaging of the neck and aorta in the cranio-caudal direction. The scanning parameters were as follows: tube voltage, 100 kV; pitch, 3.0; slice collimation, 2 × 128 × 0.6 mm by means of a z-flying focal spot; and reference tube current, 300 mAs per rotation. For all scans, patients were in a supine position with both arms raised. Each patient received an injection of 70 ml of iopromide (Ultravist 370, 370 mg I/ml; Bayer Schering Pharma, Berlin, Germany) at a flow rate of 5 ml/s, followed by 40 ml of saline solution. Bolus tracking was performed in the suprarenal descending aorta with an attenuation threshold of 100 HU. Raw data were transferred to an external workstation (syngo MMWP VE 36 A; Siemens Healthcare, Forchheim, Germany) for further postprocessing. Routine brain CT plain scans with 5-mm slice thickness were performed on postoperative patients refusing brain MRI due to stent implantation.

### Preoperative Cerebral DWI Imaging

All patients with ATAAD underwent MRI examination of DWI. GE Signa 1.5 Tesla superconducting scanner with a gradient field of 200 mT/m was used. Echo-planar imaging sequence of single excitation was used in DWI, and the diffusion sensitivity coefficient B was 1,000 s/mm^2^. The pulse repetition time/echo time, matrix, and layer thickness were 10,000/20 ms, 128 × 128, and 5 mm, respectively.

### CTA Findings, DWI Characteristics, Definitions, and Parameters

The CTA signs of ATAAD in the carotid arteries, ascending aorta (aAO), and aortic arch were selected for analysis. The aAO diameter was defined as the diameter of aAO at the largest layer. The ratio of the diameters was calculated by dividing the diameter of the true lumen by the aAO diameter. The low density of the false lumen in aAO referred to a low CT attenuation present in the false lumen of the compromised aAO. Intimal flap plaque was defined as the intimal flap thickened ≥3 mm or atherosclerotic plaque that was detected in the intimal flap of the aAO and/or aortic arch. Retrograde aAO dissection with thrombosis of the false lumen was a primary intimal tear in the aortic arch or descending aorta in which the intimal flap extended to the aAO in a retrograde manner without re-entry. Coronary artery involvement included the coronary artery originating from the false lumen of aAO or intimal flap involving the coronary artery. Lower density of a unilateral internal carotid artery (ICA) was defined as visually lower density of unilateral ICA, in which the mean CT value is more than 100 HU lower than offside ICA. For patients with ATAAD in the preoperative DWI (+) group, detailed lesion characteristics of acute cerebral infarction in DWI images were recorded and analyzed. The characteristics included the cerebral location of lesions, distribution of lesions in lobes, number of lesions, sum of the lesion area, mean diameter of all lesions, and longest diameter of the largest lesion in each patient. All patients' aortic CTA images and DWI images were analyzed by two experienced radiologists in a double-blind manner. For the quantitative data, interobserver agreement for the measurement from the 2 readers was analyzed by calculation of the intraclass correlation coefficient (ICC). ICCs are considered to be excellent if >0.74. The measurements by the two observers for each patient were for further analysis. For the qualitative data, the 2 readers always reach for the agreement and disagreements between two readers were solved by consensus.

### Statistical Analysis

Statistical analysis was performed using IBM SPSS Statistics for Windows, version 19.0 (IBM Corp., Armonk, NY, USA). Continuous values are presented as mean ± standard deviation (SD). Categorical data are presented as percentage unless otherwise specified. Continuous variables with normal data distributions were compared using independent sample *t*-test, and for data with skewed distributions, non-parametric Mann-Whitney *U*-test was used. Categorical variables were compared using χ^2^ statistics, and Fisher's exact test was used if observed frequencies were <5. Factors with a univariable value of *P* < 0.05 were incorporated into the multivariable model, and multivariate logistic regression analysis was performed to identify risk factors for postoperative new stroke. Receiver operating curves were analyzed to assess the best cut-off value of the continuous variables to predict postoperative stroke with maximal accuracy using MedCalc Software. The optimal cut-off value was calculated using the Youden index. *P* < 0.05 were considered statistically significant for all analyses.

## Results

### Patient Characteristics

Out of the 174 patients with ATAAD initially studied, 42 (24.1%) had acute cerebral infarction on preoperative DWI imaging and 32 (18.4%) had postoperative new stroke. A significantly higher percentage of postoperative new stroke was found in the DWI (+) patient group (31.0%, 13/42) than in the DWI (–) group (14.4%, 19/132, *P* = 0.016). In the DWI (–) group, statistically significant differences were noted in the incidence of hypotension (26.3% vs. 4.4%, *P* = 0.001) and the aortic cross-clamp time (113.1 ± 35.3 min vs. 95.6 ± 19.0 min, *P* = 0.002) between patients with and those without postoperative stroke. No significant difference was found between the two subgroups with regard to the rest of the baseline characteristics and intraoperative information in the DWI (+) or DWI (–) group (all *P* > 0.05). All details are listed in Table 1.

**Table 1:** Clinical characteristics of all patients with ATAAD.

**Variables**	**Overall (*n* = 174)**	**Preoperative DWI (+) (*****n*** **=** **42)**			**Preoperative DWI (–) (*****n*** **=** **132)**		
		**Postoperative stroke (+) (*n* = 13)**	**Postoperative stroke (–) (*n* = 29)**	**P**	**Postoperative stroke (+) (*n* = 19)**	**Postoperative stroke (–) (*n* = 113)**	***P***
Age (years)	48.5 ± 10.7	49.5 ± 8.9	51.0 ± 8.8	0.604	46.7 ± 11.7	48.0 ± 11.2	0.634
Male, *n* (%)	137 (78.7)	9 (69.2)	21 (72.4)	1.000	18 (94.7)	89 (78.8)	0.184
Hypertension, *n* (%)	110 (63.2)	10 (76.9)	20 (69.1)	0.874	11 (57.9)	69 (61.1)	0.794
Marfan syndrome, *n* (%)	2 (1.1)	0 (0)	1 (3.4)	1.000	0 (0)	1 (0.9)	1.000
Diabetes mellitus, *n* (%)	2 (1.1)	0 (0)	1 (3.4)	1.000	0 (0)	1 (0.9)	1.000
Dyslipidemia, *n* (%)	110 (63.2)	9 (69.2)	19 (65.5)	0.813	12 (63.2)	70 (61.9)	0.920
Coronary heart disease, *n* (%)	11 (6.3)	0 (0)	3 (10.3)	0.540	3 (15.8)	5 (4.4)	0.161
COPD, *n* (%)	2 (1.1)	0 (0)	1 (3.4)	1.000	0 (0)	1 (0.9)	1.000
Chest pain, *n* (%)	65 (37.4)	5 (38.5)	10 (34.5)	1.000	10 (52.6)	40 (35.4)	0.152
Back pain, *n* (%)	22 (12.6)	2 (15.4)	4 (13.8)	1.000	1 (5.3)	15 (13.3)	0.542
Abdominal pain, *n* (%)	32 (18.4)	4 (30.8)	4 (13.8)	0.384	4 (21.1)	20 (17.7)	0.977
Syncope, *n* (%)	4 (2.3)	1 (7.7)	2 (6.9)	1.000	0 (0)	1 (0.9)	1.000
Aortic insufficiency, *n* (%)	31 (17.8)	5 (38.5)	5 (17.2)	0.271	2(10.5)	19 (16.8)	0.723
Ejection fraction <40%	9 (5.2)	2 (15.4)	1 (3.4)	0.222	1 (5.3)	5 (4.4)	1.000
Hypotension, *n* (%)	16 (9.2)	2 (15.4)	4 (13.8)	1.000	5 (26.3)	5 (4.4)	0.004*
Mean SBP (mmHg)	132.3 ± 28.1	133.7 ± 35.0	130.0 ± 32.3	0.738	129.9 ± 26.3	133.1 ± 26.7	0.630
Mean DBP (mmHg)	70.7 ± 18.3	69.2 ± 17.1	68.4 ± 21.9	0.911	68.3 ± 16.5	72.0 ± 17.9	0.402
Aortic root replacement, *n* (%)	113 (64.9)	11 (84.6)	23 (79.3)	1.000	13 (68.4)	97 (85.8)	0.121
Total arch replacement, *n* (%)	168 (96.6)	13 (100)	28 (96.6)	1.000	19 (100)	108 (95.6)	1.000
Adjunctive coronary artery bypass, *n* (%)	17 (9.8)	2 (15.4)	2 (6.9)	0.766	3 (15.8)	10 (8.8)	0.601
Aortic cross-clamp time (min)	100.0 ± 23.3	105.4 ± 24.8	105.9 ± 24.7	0.951	113.1 ± 35.3	95.6 ± 19.0	0.002*
LBI time (min)	29.3 ± 6.3	30.0 ± 6.3	28.9 ± 5.9	0.761	30.6 ± 5.6	29.2 ± 6.6	0.421

Results are presented as n (%) or mean ± SD; *P-value based on the non-parametric Mann-Whitney test; COPD, chronic obstructive pulmonary disease; SBP, systolic blood pressure; DBP, diastolic blood pressure; LBI, lower body ischemia.

### CTA Findings of Preoperative DWI (–) Related to Postoperative New Stroke in Patients With ATAAD

The measurements by the two observers achieved good consistency with all ICC >0.74. Patients with ATAAD with postoperative new stroke had a significantly higher percentage of retrograde aAO dissection with thrombosis of the false lumen (26.3 vs. 7.1%, *P* = 0.009), the aortic arch entry (68.4 vs. 43.4%, *P* = 0.043), and dissection involving the coronary artery (47.4 vs. 6.2%, *P* = 0.000) than the patients without postoperative stroke in the preoperative DWI (–) cohort. Statistically significant differences were not found between the two subgroups with or without postoperative stroke with regard to the other CTA findings (all *P* > 0.05). Detailed results are shown in Table 2.

**Table 2:** CTA findings in preoperative DWI (–) patients with or without postoperative stroke.

**CTA findings**	**Preoperative DWI (–)**		
	**Postoperative stroke (+) (*n* = 19)**	**Postoperative stroke (–) (*n* = 113)**	***P***
aAO diameter (mm)	46.8 ± 7.8	47.8 ± 6.9	0.586
Ratio of the diameters, *n* (%)	0.4 ± 0.2	0.4 ± 0.2	0.239
Low density of the false lumen in aAO, *n* (%)	6 (31.6)	41 (36.3)	0.692
Intimal flap plaque, *n* (%)	6 (31.6)	21 (18.6)	0.321
Retrograde aAO dissection with thrombosis of the false lumen, *n* (%)	5 (26.3)	8 (7.1)	0.029*
Entry in the aAO, *n* (%)	6 (31.6)	62 (54.9)	0.060
Size of aAO entry (mm)	11.5 ± 7.1	15.7 ± 8.3	0.235
Aortic arch entry, *n* (%)	13 (68.4)	49 (43.4)	0.043*
Size of aortic arch entry (mm)	12.8 ± 6.8	11.0 ± 9.7	0.694
Coronary artery involvement, *n* (%)	9(47.4)	7(6.2)	0.000*
CCA dissection, *n* (%)	7 (36.8)	30 (26.5)	0.355
CCA originating from the false lumen, *n* (%)	1 (5.3)	3 (2.7)	0.467
Lower density of unilateral ICA, *n* (%)	0 (0)	7 (6.2)	0.574
VA originating from the false lumen, *n* (%)	1 (5.3)	2 (1.8)	0.375
VA originating from the aortic arch, *n* (%)	0 (0)	3 (2.7)	1.000
SA dissection, *n* (%)	5 (26.3)	15 (13.3)	0.262
SA originating from the false lumen, *n* (%)	0 (0)	1 (0.9)	1.000

Results are presented as n (%) or mean ± SD; *P-value based on the **non-parametric Mann-Whitney test**; aAO, ascending aorta; CCA, common carotid artery; ICA, internal carotid artery; VA, vertebral artery; SA, subclavian artery.

### CTA Findings and DWI Characteristics of Preoperative DWI (+) Related to Postoperative New Stroke in Patients With ATAAD

Table 3 lists the CTA findings and DWI features in the DWI (+) group. The only significantly different CTA finding between the two subgroups was the aortic arch entry (76.9 vs. 41.4%, *P* = 0.033). Patients with postoperative new stroke had significantly higher percentages of cerebral lesions distributed ≥3 lobes in the unilateral hemisphere (69.2 vs. 6.9%, *P* = 0.000) and total lesion number >5 (46.2 vs. 10.3%, *P* = 0.027) than the subjects without postoperative stroke. All 9 cases of DWI lesions distributed ≥3 lobes in the unilateral hemisphere were present in the right hemisphere. Out of the 9 patients, 5 had right common carotid artery (CCA) dissection and 1 had bilateral CCA dissection. The remaining 3 patients had no dissection in any of the supraaortic branches, but had retrograde aAO dissection with thrombosis of the false lumen, intimal flap plaque, or preoperative hypotension.

**Table 3:** CTA findings and DWI characteristics in preoperative DWI (+) patients with or without postoperative stroke.

**Imaging findings**	**Preoperative DWI (+)**		
	**Postoperative stroke (+) (*n* = 13)**	**Postoperative stroke (–) (*n* = 29)**	***P***
**CTA findings**			
aAO diameter (mm)	47.1 ± 6.6	47.9 ± 6.1	0.684
Ratio of the diameters	0.2 ± 0.1	0.3 ± 0.1	0.444
Low density of the false lumen in aAO, *n* (%)	4 (30.8)	9 (31.0)	1.000
Intimal flap plaque, *n* (%)	3 (23.1)	9 (31.0)	0.874
Retrograde aAO dissection with thrombosis of the false lumen, *n* (%)	2 (15.4)	4 (13.8)	1.000
Entry in the aAO, *n* (%)	3 (23.1)	16 (55.2)	0.053
Size of aAO entry (mm)	12.7 ± 4.9	15.6 ± 9.5	0.620
Aortic arch entry, *n* (%)	10 (76.9)	12 (41.4)	0.033*
Size of the aortic arch entry (mm)	11.0 ± 4.2	9.8 ± 4.1	0.745
Coronary artery involvement, *n* (%)	4 (30.8)	9 (31.0)	1.000
CCA dissection, *n* (%)	10 (76.9)	17 (58.6)	0.426
CCA originating from the false lumen, *n* (%)	0 (0)	1 (3.4)	1.000
Lower density of unilateral ICA, *n* (%)	3 (23.1)	2 (6.9)	0.326
VA originating from the false lumen, *n* (%)	1 (7.7)	1 (3.4)	0.5281
VA originating from the aortic arch, *n* (%)	1 (7.7)	0 (0)	0.310
SA dissection, *n* (%)	2 (15.4)	7 (24.1)	0.816
SA originating from the false lumen, *n* (%)	0 (0)	1 (3.4)	1.000
Lesion characteristics in cerebral DWI			
Location of lesions			
Supratentorial, *n* (%)	9 (69.2)	19 (65.5)	1.000
Infratentorial, *n* (%)	1 (7.7)	1 (3.4)	1.000
Supratentorial + infratentorial, *n* (%)	3 (23.1)	9 (31.0)	0.874
Left hemisphere, *n* (%)	0 (0)	1 (3.4)	1.000
Right hemisphere, *n* (%)	7 (53.8)	17 (58.6)	0.773
Bilateral hemisphere, *n* (%)	6 (46.2)	11 (37.9)	0.616
Gray matter, *n* (%)	5 (38.5)	19 (65.5)	0.101
White matter, *n* (%)	3 (23.1)	5 (17.2)	0.984
Gray matter + white matter, *n* (%)	5 (38.5)	6 (20.7)	0.406
Distribution of lesions in lobes			
Number of involved lobes	4.5 ± 2.8	3.0 ± 2.0	0.098
<3 lobes in the bilateral hemisphere, *n* (%)	0 (0)	5 (17.2)	0.302
≥3 lobes in the bilateral hemisphere, *n* (%)	1 (7.7)	7 (24.1)	0.407
<3 lobes in the unilateral hemisphere, *n* (%)	3 (23.1)	15 (51.7)	0.083
≥3 lobes in the unilateral hemisphere, *n* (%)	9 (69.2)	2 (6.9)	<0.001*
Number of lesions	6.0 ± 5.1	3.0 ± 2.6	0.064
<3, *n* (%)	4 (30.8)	18 (62.1)	0.060
3–5, *n* (%)	3 (23.1)	7 (24.1)	1.000
>5, *n* (%)	6 (46.2)	3 (10.3)	0.027*
Sum of the lesion area (mm^2^)	370.1 ± 557.4	158.7 ± 382.1	0.160
Mean diameter of all lesions (mm)	3.1 (2.6-5.8)	2.8 (2.4-3.0)	0.109
Longest diameter of the largest lesion (mm)	3.4 (2.9-6.8)	3.2 (3.0-3.65)	0.346

Results are presented as n (%) or mean ± SD; *P-value based on the non-parametric Mann-Whitney test; aAO, ascending aorta; CCA, common carotid artery; ICA, internal carotid artery; VA, vertebral artery; SA, subclavian artery.

### Preoperative Risk Predictors for Postoperative New Stroke in Patients With ATAAD

**Figure 1:**
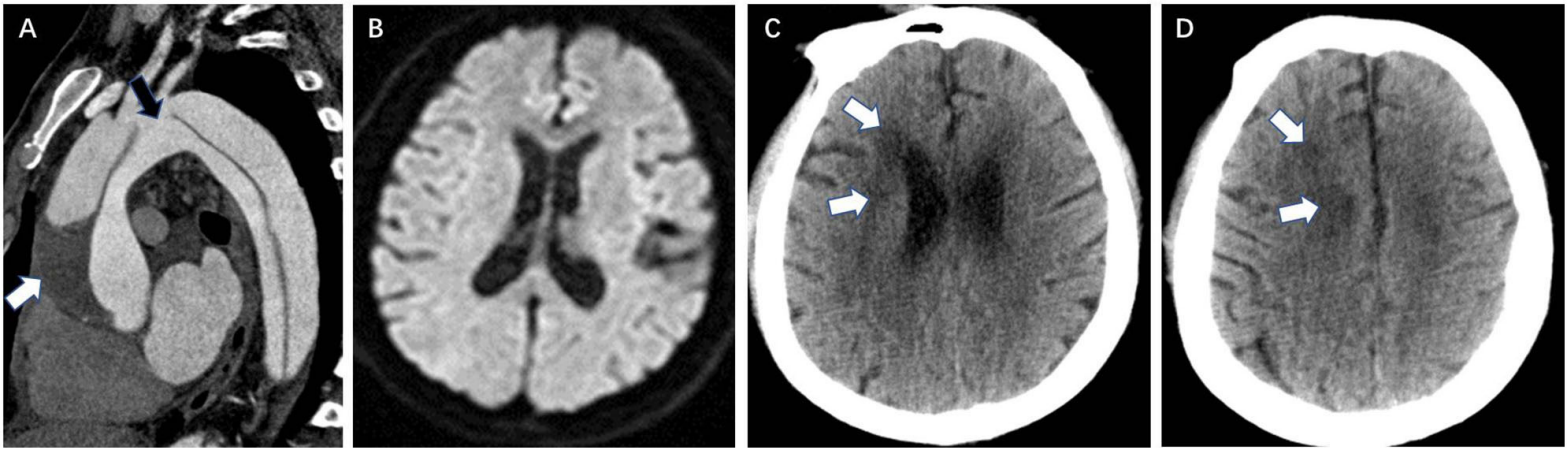
Retrograde aAO dissection with thrombosis of the false lumen is a CTA risk finding for postoperative new stroke in a 39-year-old man with ATAAD. **(A)** Oblique sagittal MPR image shows an entry tear (black arrow) in the aortic arch and the intimal flap extends into the aAO in a retrograde manner. Low-density thrombus (white arrow) is present in the false lumen due to the integrated intimal fap in the aAO. **(B)** Preoperative diffusion-weighted magnetic resonance images show normal brain. **(C,D)** Brain CT 13 days after surgery shows a new low-density ischemic infarction in the right frontal lobe, area around the right ventricular body, and centrum semiovale (white arrow). aAO, ascending aorta; CTA, computed tomography angiography; ATAAD, acute type A aortic dissection; MPR, multiplanar reformation.

**Figure 2:**
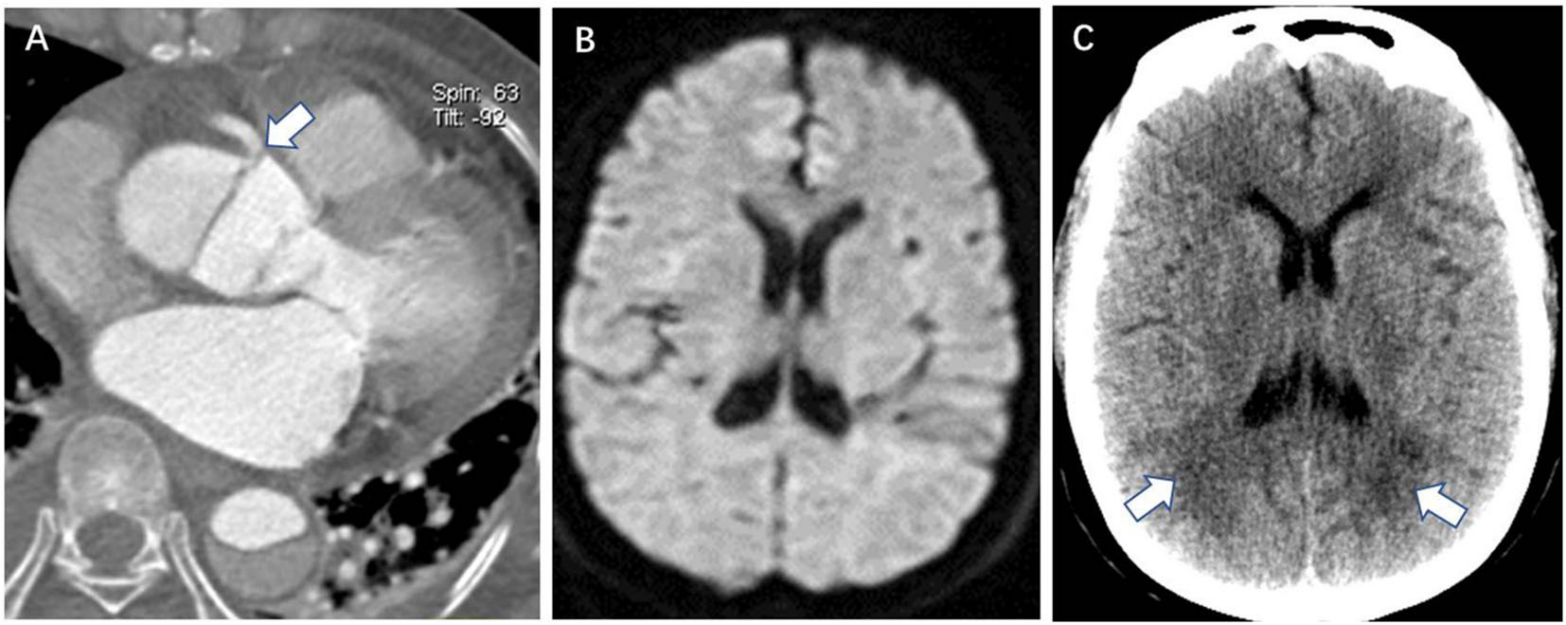
Coronary artery involvement is a CTA risk finding for postoperative new stroke in a 32-year-old man with ATAAD. **(A)** Axial image shows the intimal flap involving the origin of the right coronary artery (white arrow). **(B)** Preoperative diffusion-weighted magnetic resonance image shows the normal brain. **(C)** Brain CT 11 days after surgery shows a new low-density ischemic infarction in the bilateral deep occipital lobes (white arrow). CTA, computed tomography angiography; ATAAD, acute type A aortic dissection.

**Figure 3:**
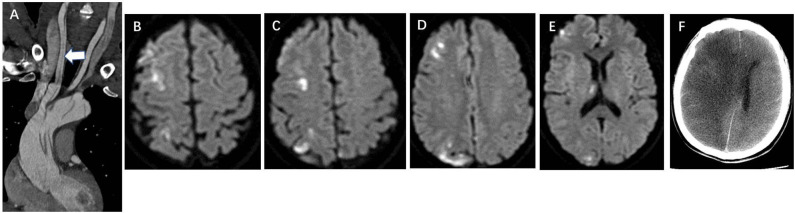
Cerebral infarction lesions distributed ≥3 lobes in the unilateral hemisphere is a DWI risk finding for postoperative new stroke in a 45-year-old man with ATAAD. **(A)** CTA oblique coronal MPR image shows aAO and aortic arch dissection involving whole right CCA (white arrow). **(B–E)** Preoperative diffusion-weighted magnetic resonance image shows multiple high signal of cerebral infarctions distributed in the frontal lobe, parietal lobe, occipital lobe, and thalamus in different layers of the right hemisphere. **(F)** Brain CT 20 days after surgery shows a massive cerebral infarction in the right hemisphere. DWI, diffusion-weighted magnetic resonance imaging; ATAAD, acute type A aortic dissection; CTA, computed tomography angiography; MPR, multiplanar reformation; aAO, ascending aorta; CCA, common carotid artery.

In the DWI (–) group, multivariate logistic regression analysis identified that the retrograde aAO dissection with thrombosis of the false lumen (OR = 7.981, *P* = 0.010) (Figure 1), the aortic arch entry (OR = 5.099, *P* = 0.035), dissection involving the coronary artery (OR = 10.787, *P* = 0.001) (Figure 2), and hypotension (OR = 22.779, *P* = 0.002) were independent risk predictors for postoperative new stroke in patients with ATAAD (Table 4). In the DWI (+) group, only cerebral lesions distributed ≥3 lobes in the unilateral hemisphere (OR = 16.509, *P* = 0.007) (Figure 3) were identified as an independent risk factor for patients with ATAAD with postoperative new stroke (Table 4).

**Table 4:** Risk factors for postoperative new stroke in patients with ATAAD.

**Variable**	**OR**	**95% CI**	***P*-value**
**DWI (–)**			
Retrograde aAO dissection with thrombosis of the false lumen	7.981	1.657–38.445	0.010*
Aortic arch entry	5.099	1.124–23.132	0.035*
Coronary artery involvement	10.787	2.544–45.743	0.001*
Hypotension	22.779	3.065–169.300	0.002*
Aortic cross-clamp time (min)	1.044	1.000–1.089	0.050
**DWI (+)**			
Aortic arch entry	2.885	0.410–20.297	0.287
Lesions distributed ≥3 lobes in the unilateral hemisphere	16.509	2.169–125.662	0.007*
Number of lesions >5	2.738	0.280–26.764	0.386

*aAO, ascending aorta; CI, confidence interval; OR, odds ratio*.

* *P-values based on multivariate logistic regression analysis*.

## Discussion

The CTA and DWI risk findings for postoperative new stroke in patients with ATAAD were totally different in the DWI (+) or DWI (–) group. Multivariate analysis showed that the retrograde aAO dissection with thrombosis of the false lumen, the aortic arch entry, the coronary artery involvement, and preoperative hypotension were independent risk predictors for postoperative stroke in patients with ATAAD with normal brain. In patients with ATAAD with preoperative cerebral infarction in DWI, only lesions distributed ≥3 lobes in the unilateral hemisphere were an independent risk factor for postoperative new stroke.

Preoperative cerebral malperfusion in patients with ATAAD was a predictor for postoperative stroke and detrimental outcome (6). However, assessment of cerebral malperfusion may not be accurate only based on symptoms and signs as well as the dissection of the supraaortic branches without intracranial imaging evidence, which may further lead to unreliable prediction of postoperative stroke. The DWI may be the best imaging modality to assess cerebral infarction. In case of emergency, however, fewer patients with ATAAD undergo this test prior to surgery. Therefore, few studies may obtain direct evidence of brain abnormalities before surgery in patients with ATAAD. All patients with ATAAD in this study underwent preoperative cranial DWI and postoperative CT. Evaluation of cerebral infarction resulting from cerebral hypoperfusion prior to surgery and postoperative new stroke is accurate. Evaluation of preoperative risk factors for postoperative stroke based on imaging information is also accurate.

In this study, preoperative acute cerebral infarction resulting from cerebral hypoperfusion is frequent and is found in 24.1% of the brains, which is higher than the results in previous studies (17–19). This may be related to the fact that the majority of the patients with ATAAD in this study had neurological symptoms and signs. Our results, showing a percentage of 18.4% of postoperative stroke in patients with ATAAD, are similar to those reported in a previous study (1). A total of 31% (13/42) of patients with ATAAD with preoperative cerebral infarction develop postoperative new stroke, further indicating that preoperative cerebral malperfusion highly correlated with postoperative stroke. Moreover, it is perhaps noteworthy that 14.4% (19/174) of the patients with ATAAD with normal brain also presented postoperative stroke.

In the DWI (–) cohort, our data suggest that the retrograde aAO dissection with thrombosis of the false lumen, the aortic arch entry, and dissection involving the coronary artery, as well as preoperative hypotension are independent risk predictors for postoperative stroke in patients with ATAAD. Previous studies indicated that hypotension and aortic cross-clamp time are predictors for postoperative stroke and neurological dysfunction (6, 20, 21). Unexpectedly, aortic cross-clamp time is not an independent risk predictor for postoperative stroke in this study, but it is significantly longer in patients with ATAAD with postoperative stroke (113.1 ± 35.3 min) than in subjects without stroke (95.6 ± 19.0 min, *P* = 0.002). Our previous study confirmed that retrograde aAO dissection is an independent predictor of postoperative permanent neurological dysfunction (PND) in patients with ATAAD. This condition is characterized by an entry tear in the aortic arch and descending aorta, and thrombus in the false lumen owing to the integrated intimal flap in aAO. The present study further accurately confirms that the retrograde aAO dissection with thrombosis of the false lumen is one of the risk predictors for postoperative stroke in patients with ATAAD with normal brain, but not in those with infarcted brain. The key point of this CTA finding was the thrombus in the false lumen, and almost all of the patients with retrograde aAO dissection had thrombus in the false lumen. This imaging finding implies a high probability of thrombus flowing into the brain

brain via aortic arch tear during surgical procedure. Thus, particular attention should be given to this imaging finding, which may apprise surgeons of meticulous attention to critical procedural details and avoid the potential risk of postoperative stroke owing to thrombus falling off. The aortic arch entry as a risk factor for postoperative stroke is not unexpected. The potential cause may be similar to the CTA finding of retrograde aAO dissection with thrombosis of the false lumen. An aortic arch entry may enable the pre- and intraoperative microthrombus flow more easily into the craniocervical arteries via the tear point, resulting in subsequent cerebral artery occlusion and cerebral infarction. The CTA finding of dissection involving the coronary artery as a risk factor for postoperative stroke is unexpected. Coronary artery bypass grafting is associated with postoperative neurological complications, including stroke, with an incidence of 2.6–7.6% (22, 23). Thus, one might speculate that postoperative stroke might be associated with longer surgical time due to additional coronary artery bypass grafting.

Although the distribution characteristics of preoperative cerebral lesions are rarely described in previous reports of ATAAD, this study demonstrates that cerebral lesions distributed ≥3 lobes in the unilateral hemisphere and number of cerebral lesions >5 were significantly more common in patients with ATAAD with postoperative stroke in the DWI (+) group. Cerebral lesions distributed ≥3 lobes in the unilateral hemisphere were also an independent predictor for postoperative new stroke in patients with ATAAD with preoperative cerebral lesions. It is perhaps noteworthy that all 9 cases of lesions distributed ≥3 lobes in the unilateral hemisphere were in the right hemisphere, which may be due to right CCA dissection that was observed in 6 cases. The presence of DWI finding of cerebral lesions ≥3 lobes in the unilateral hemisphere implies that the lesions are multiple and widely distributed, with more possibility of cerebral hypoperfusion leading to postoperative new stroke. More than 5 infarction lesions in the brain, may be localized in one lobe, despite the high number. These imaging findings may help to individualize the optimized surgical strategy including brain protection techniques. For patients with ATAAD with preoperative cerebral lesions ≥3 lobes in the right hemisphere but without information of the circle of Willis, an anterograde bilateral selective cerebral perfusion may be better than unilateral cerebral perfusion.

This study has several limitations. First, it was a retrospective study with selection bias, and the follow-up period was limited. Second, this is a single-center data with limited preoperative DWI samples and limited number of postoperative new stroke. Larger multicenter studies may be necessary to determine the real imaging risk findings for postoperative new stroke in patients with ATAAD with different preoperative status. Third, preoperative cerebral DWI examination was not often available in emergency situations. However, analysis based on imaging presence or absence of cerebral infarction may provide more accurate evaluation of preoperative risk factors for postoperative new stroke in patients with ATAAD with normal or abnormal neurological status.

In conclusion, for patients with ATAAD with normal brain, in addition to preoperative hypotension, CTA findings of false lumen thrombosis and aortic arch entry may remind surgeons of critical procedural details to avoid thrombus falling off and entering the brain through the tear point. For patients with ATAAD with preoperative neurological symptoms and signs, a cerebral DWI may be necessary and infarction lesions distributed ≥3 lobes in the unilateral hemisphere may suggest a high possibility of postoperative new stroke.

## Data Availability Statement

The raw data supporting the conclusions of this article will be made available by the authors, without undue reservation.

## Ethics Statement

The studies involving human participants were reviewed and approved by the institutional review board of Xijing Hospital affiliated with the Fourth Military Medical University. The patients/participants provided their written informed consent to participate in this study. Written informed consent was obtained from the individual(s) for the publication of any potentially identifiable images or data included in this article.

## Author Contributions

HZ performed conception and design of the paper, the patient data acquisition and analysis, and the draft the article. FG joined conception and design of the paper and the draft as well as the revise of the article. JX, YZ, and DW performed acquisition, analysis, or interpretation of data. WD performed the revision of the article. MZ performed the conception and design of the paper and the revision of the article. All authors read and approved the final manuscript.

## Conflict of Interest

The authors declare that the research was conducted in the absence of any commercial or financial relationships that could be construed as a potential conflict of interest.
